# Generation of a Fibrin Based Three-Layered Skin Substitute

**DOI:** 10.1155/2015/170427

**Published:** 2015-07-07

**Authors:** Johanna Kober, Alfred Gugerell, Melanie Schmid, Lars-Peter Kamolz, Maike Keck

**Affiliations:** ^1^Division of Plastic and Reconstructive Surgery, Department of Surgery, Medical University of Vienna, Waehringer Guertel 18-20, 1090 Vienna, Austria; ^2^Division of Plastic, Aesthetic and Reconstructive Surgery, Department of Surgery, Medical University Graz, Auenbruggerplatz 29, 8036 Graz, Austria

## Abstract

A variety of skin substitutes that restore epidermal and dermal structures are currently available on the market. However, the main focus in research and clinical application lies on dermal and epidermal substitutes whereas the development of a subcutaneous replacement (hypodermis) is often disregarded. In this study we used fibrin sealant as hydrogel scaffold to generate a three-layered skin substitute. For the hypodermal layer adipose-derived stem cells (ASCs) and mature adipocytes were embedded in the fibrin hydrogel and were combined with another fibrin clot with fibroblasts for the construction of the dermal layer. Keratinocytes were added on top of the two-layered construct to form the epidermal layer. The three-layered construct was cultivated for up to 3 weeks. Our results show that ASCs and fibroblasts were viable, proliferated normally, and showed physiological morphology in the skin substitute. ASCs were able to differentiate into mature adipocytes during the course of four weeks and showed morphological resemblance to native adipose tissue. On the surface keratinocytes formed an epithelial-like layer. For the first time we were able to generate a three-layered skin substitute based on a fibrin hydrogel not only serving as a dermal and epidermal substitute but also including the hypodermis.

## 1. Introduction

Soft tissue damage following trauma or tumor resection often results in an exigent need of highly sophisticated, complex tissue substitutes. Due to limited donor site for tissue reconstruction (flaps) and morbidity in large scale defect reconstruction, plastic surgeons often reach their limitations in reconstructing these defects. New biomaterials and improved scaffold processing techniques have been developed over the last years, and novel promising scaffold materials can be fabricated and offered to the patients [1]. However, the main focus in research and clinical application lies on substitutes consisting of the dermal and epidermal layer [2–4] whereas the development of a subcutaneous replacement (hypodermis) is often disregarded. Particularly in deep dermal burns such constructs would be of an enormous advantage.

The skin is composed of three layers. The hypodermis mainly consists of adipocytes, fibroblasts, and adipose-derived stem cells and protects the body from stress and strain. Above the hypodermis lies the dermis which is mainly composed of fibroblasts and different extracellular matrix proteins such as collagen, elastin, and glycosaminoglycans. The outermost layer of the skin is called epidermis and consists of keratinocytes and only sparse extracellular matrix.

The ability to “engineer” a three-dimensional skin construct to restore a physical deformity without the need for multiple surgical steps and a painful, scarred donor site remains a primary goal of scientists and plastic surgeons. The substitute should incorporate a biocompatible scaffold that defines the appropriate three-dimensional tissue architecture of adequate size to have clinical applicability to current reconstructive problems and promotes host integration and implant vascularization [5]. Ultimately, the construct should demonstrate certain stability over time but also be biodegradable as it is replaced by healthy host soft tissue. Mechanical properties are particularly important, and, ideally, the scaffold mimics the native tissue into which it will be incorporated [6]. On a macroscopic level, this is important to ensure that the implant has a natural feel and to minimize inflammation (reaction on artificial materials) and scar tissue formation. However, recent research has also highlighted the importance of mechanical properties on a cellular level, with the substrate stiffness dramatically influencing the differentiation response of seeded cells [7].

In this study we used fibrin sealant as hydrogel scaffold to generate the three-dimensional skin substitute. Fibrin is an established material in surgical applications and was shown to be highly biocompatible in tissue engineering and cell delivery [8–10]. It has already been used as matrix for generating skin substitutes in vitro [11] and in vivo [12] and was suggested by Peterbauer-Scherb et al. to represent a suitable scaffold material for adipose tissue formation [13].

The hypodermis mainly consists of mature adipocytes but also other cell types such as adipose-derived stem cells (ASCs). These precursor cells are located between mature adipocytes. They can serve as an ideal autologous cell source for adipose tissue engineering approaches, since they are more resistant to mechanical damage and ischemia than mature adipocytes [14]. Adipocytes and ASCs can easily be harvested during liposuction or resection of adipose tissue. ASCs have been shown to proliferate rapidly and differentiate into bone, adipogenic, and chondrogenic lineage both in vitro and in vivo and are known to migrate to injured sites [15–18].

Aim of the present study was to generate a three-layered skin substitute consisting of an epidermal, dermal, and a hypodermal tissue layer. Human ASCs and mature adipocytes were used to construct the hypodermis.

## 2. Materials and Methods

### 2.1. Patients and Tissue Harvesting

Skin and fat tissue were gained from otherwise healthy patients undergoing body contouring surgery. All subjects gave written informed consent before participating in the study which has been approved by the ethics committee of the Medical University of Vienna and the General Hospital Vienna (EK number 1949/2012).

### 2.2. Isolation of Human Adipose-Derived Stem Cells and Adipocytes

Adipose tissue was washed in PBS (phosphate buffered saline, PAA Laboratories GmbH, Pasching, Austria) and ASCs and mature adipocytes were isolated as described before [19]. Briefly, the adipose tissue was minced and digested with 2 mg/mL Collagenase Type IV (Sigma-Aldrich, St. Louis, MO, USA) in Hanks' buffered salt solution (HBSS, Lonza, Walkersville, MD, USA) for 1 h at 37°C with constant shaking. The stromal vascular fraction (SVF) was isolated by centrifugation and red blood cells were lysed in 2 mL Red Blood Cell Lysing Buffer (Sigma-Aldrich). Cells were centrifuged and resuspended in culture medium DMEM (Gibco, Life Technologies Ltd., Paisley, UK) supplemented with 10% fetal calf serum (Hyclone, Fisher Scientific GmbH, Schwerte, Germany), 100 units/mL penicillin, and 100 *μ*g/mL streptomycin (Life Technologies Ltd., Paisley, UK). Isolated cells were counted in a Bürker-Türk counting chamber (Hecht Assistant, Sondheim, Germany) with Trypan Blue stain (0.4%, Gibco, Life Technologies Ltd., Paisley UK) to prove viability. By cultivation of SVF, selection and expansion of ASCs were achieved. ASCs were cultured as a monolayer at 37°C in supplemented proliferation/cell culture medium in a humidified atmosphere with 5% CO_2_. For all experiments ASC passages up to passage 2 were used.

Freshly isolated adipocytes were collected during the isolation process from the adipose phase after the first centrifugation step. Adipocytes were washed two times with PBS to remove collagenase and were used for experiments right away.

### 2.3. Isolation of Human Dermal Fibroblasts

Human donor skin was cut into small pieces, put in 6-well culture plate, and incubated for 14 days in culture medium DMEM supplemented with 10% fetal calf serum, 1% glutamine (Gibco, Life Technologies Ltd.), 100 units/mL penicillin, and 100 *μ*g/mL streptomycin. Fibroblasts growing out onto the bottom of the 6-well culture plate were cultured upon confluence and were split for further cultivation in culture flasks.

### 2.4. Human Keratinocytes

Keratinocytes (Evercyte, Vienna, Austria) were cultured in DermaLife K Complete Medium (CellSystems, Troisdorf, Germany).

### 2.5. Construction of a Three-Layered Skin Substitute with a Fibrin Hydrogel Matrix

Fibrinogen and thrombin (both Baxter AG, Vienna, Austria) were reconstituted according to the manufacturer's protocol. In brief, lyophilized sealer protein component was reconstituted in 3,000 KIE/mL aprotinin solution; the lyophilized thrombin component was reconstituted in 40 mM CaCl_2_ solution. The components were diluted in PBS to a concentration of 25 mg/mL fibrinogen and 2.5 IU/mL thrombin as end concentration in the clot. For fibrin clot formation, equal volumes of fibrinogen and thrombin components were mixed in ThinCert cell culture inserts (pore size 8 *μ*m, Greiner bio-one, Frickenhausen, Germany) and clots were incubated at 37°C for 20 minutes for polymerization.

For the hypodermal layer 3 × 10^5^ ASCs and 2.5 × 10^4^ adipocytes were embedded in 100 *μ*L fibrin clots in cell culture inserts. After 15 minutes of polymerization another clot was polymerized on top of the hypodermal layer with 3 × 10^5^ fibroblasts embedded for the construction of the dermal layer of the skin substitute. After another 15 minutes polymerization period keratinocytes (0.6–1 × 10^6^ cells/clot) were added on top of the two-layered construct for the epidermal layer. The three-layered construct was cultivated for up to 3 weeks in DMEM + 10% FCS where keratinocytes were exposed to air according to the air-liquid interface cultivation model [20].

### 2.6. Histological Evaluation of Construct

All samples were fixed overnight in 4.5% neutrally buffered formalin (SAV LP, Flintsbach a. Inn, Austria). After embedding in paraffin, 4 *μ*m cross sections were cut, deparaffinized, and stained. For morphology staining, fixed probes were stained with Mayer's Hemalaun solution (Merck KGaA, Darmstadt, Germany) and Eosin 1 wt. % (Sigma-Aldrich, St. Louis, MO, USA) or with 2.5 *μ*M/mL SYTOX Green Nucleic Acid Stain/dH_2_O (Molecular Probes, Eugene, OR, USA) under light protection. Pictures were taken with an AxioImager microscope (Zeiss, Jena, Germany).

### 2.7. Viability and Proliferation Testing

Cells (ASCs or fibroblasts: 3 × 10^5^) were embedded in the fibrin gel (100 *μ*L) and cultivated in culture medium for 24 hours before medium was discarded and proliferation of cells was measured using a CellTiter96 Non-Radioactive Proliferation Assay (Promega Corporation, Madison, WI). Fibrin clots with no cells were used as negative control. Cell number was evaluated according to manufacturer's protocol: supernatants were discarded and 45 *μ*L dye solution was added to 300 *μ*L culture medium. After 1.5 hours of incubation at 37°C in a humidified atmosphere with 5% CO_2_ stop solution was added. After one hour of incubation 100 *μ*L of the supernatants was transferred in triplicates on a 96-well plate and absorbance was measured at 555 nm on a Wallac 1420 VICTOR2 plate reader (PerkinElmer, Waltham, MA, USA). Further proliferation assays were performed on day 7 and day 14 of the experiment. Additionally, fibrin clots with no cells were used for negative control measurement. Fibrin clots with cells from at least 4 different donors were analyzed in independent experiments.

### 2.8. Adipocyte Differentiation

To assess adipocyte maturation in the fibrin clot, ASCs (3 × 10^5^/100 *μ*L) were embedded in fibrin hydrogel and 50 *μ*L clots were formed in cloning rings (Sigma-Aldrich, St. Louis, MO, USA). For 5 days clots were incubated with Preadipocyte Differentiation Medium (PromoCell GmbH, Heidelberg, Germany) before the medium was changed to Adipocyte Nutrition Medium (PromoCell GmbH, Heidelberg, Germany) which was changed every third day for the rest of the experiment. Differentiation was evaluated by fluorescent AdipoRed staining 7, 14, 21, and 28 days after induction of differentiation. Clots were incubated with AdipoRed Assay Reagent (Lonza, Walkersville, MD, USA) according to manufacturer's protocol. Fibrin clots with differentiated ASCs were analyzed using a Nikon Microphot-FXA microscope with the NIS-Elements AR 3.0 software (Nikon). Differentiation experiments for microscopic analysis were conducted with ASCs from 11 different donors.

### 2.9. Glycerol Assay

For the quantitative determination of glycerol in the supernatants of three-layered skin substitute in culture, 3 × 10^5^/100 *μ*L ASCs were embedded in 50 *μ*L fibrin clots and were incubated with Preadipocyte Differentiation Medium for 5 days before the medium was changed to Adipocyte Nutrition Medium. On days 7, 14, 21, and 28 after induction of differentiation supernatants were analyzed using a Glycerol Assay (Randox Laboratories Ltd., Crumlin, UK) to quantify the progress of ASCs differentiation. Briefly, medium was changed 24 hours before analysis. 50 *μ*L of the supernatant was transferred as triplets on 96-well plates and was mixed with 100 *μ*L reagent. Absorbance was measured at 490 nm on a Wallac 1420 VICTOR2 plate reader. Experiment was done with cells from 4 different donors. Native adipose tissue of the same weight as the 50 *μ*L fibrin clots was used as positive tissue control. Fresh adipose tissue was put in Adipocyte Nutrition Medium for 24 hours and the glycerol concentration in the supernatant was measured as described above.

### 2.10. Statistics

Statistical analysis was done with the GraphPad Prism software. Data are expressed as means ± SEM of at least 3 independent experiments. Statistical comparisons for all experimental settings were based on two-sample *t*-test with *P* < 0.05 considered as significant.

## 3. Results

### 3.1. Generation of a Three-Layered Skin Substitute with Fibrin Hydrogel Matrix

**Figure 1:**
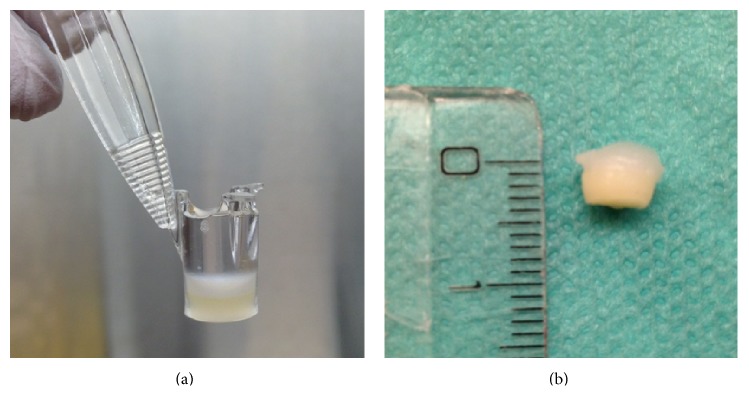
(a) Three-layered skin substitute (5 mm^3^) constructed of fibrin hydrogel for in vitro analysis. (b) Lower layer (yellow) consists of fibrin clot with ASCs and mature adipocyte as hypodermal layer. Upper layer (white) consists of hydrogel mixed with fibroblasts mimicking the dermal layer. On top keratinocytes were seeded as epidermal layer.

For the hypodermal layer ASCs and mature adipocytes were embedded in the fibrin hydrogel. On top, another fibrin clot with fibroblasts was placed for the construction of the dermal layer. Keratinocytes were added on top of the two-layered construct to form the epidermal layer. The three-layered construct was cultivated for up to 3 weeks in cell culture inserts with keratinocytes being exposed to air according to the air-liquid interface cultivation model [20]. Constructs measured 4-5 mm^3^ for the following in vitro experiments (Figure 1).

**Figure 2:**
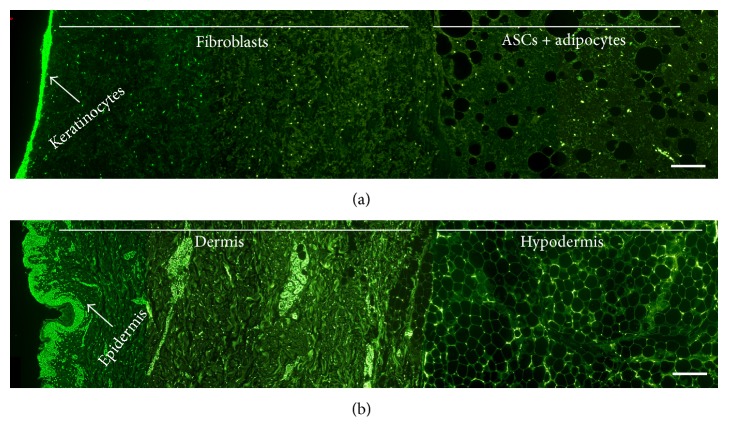
Cross sections of three-layered artificial skin show similar morphological structure to native skin. (a) Three-layered skin construct out of fibrin hydrogel stained with SYTOX Green Nucleic Acid Stain on day 4. (b) Native skin stained with SYTOX Green Nucleic Acid Stain. Bars represent 200 *μ*m.

Cross section probes of the generated artificial skin substitute (Figure 2(a)) were analyzed and showed similar hypodermal, dermal, and epidermal structures when compared to native skin (Figure 2(b)). Cells were distributed equally in the gel and showed normal morphology. On the surface keratinocytes formed an epithelial-like layer.

### 3.2. Viability of ASCs and Fibroblasts in the Three-Layered Skin Substitute

To determine if ASCs and fibroblasts are viable and proliferate at a normal rate in the fibrin construct, we incubated fibrin clots with either ASCs or fibroblasts embedded for 1 and 7 days in culture medium and analyzed the cell number (matrix w/cells). Fibrin clots without cells were used as negative control. Following incubation cells were analyzed using a cytotox assay.

**Figure 3:**
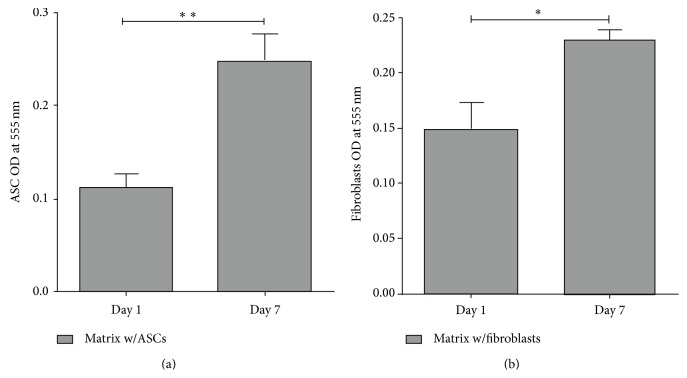
ASCs and fibroblasts are viable and proliferate in the construct. (a) ASC from six different donors (*n* = 6) and (b) fibroblasts from four donors (*n* = 4) were used in independent experiments. Data are expressed as mean ± SEM. Significant differences are indicated by asterisks: ^*∗*^ *P* < 0.05, ^*∗∗*^ *P* < 0.01.

The results showed that both cell types were viable and proliferated in the construct as the significant difference in cell number between day 1 and day 7 demonstrated in Figure 3 (ASCs: *P* < 0.01; fibroblasts: *P* < 0.05). ASCs and fibroblasts seemed to reach their highest level of proliferation on day 7 as we also tested the cell number on day 14 (data not shown).

Hence, the results show an increasing cell number of ASCs and fibroblasts in the three-layered artificial skin substitute during the first week.

### 3.3. Hypodermis Construction in Hydrogel Matrix: Differentiation Potential of ASCs

In our construct we seeded ASCs in combination with mature adipocytes to add a hypodermis to the epidermal and dermal layer of our skin substitute. However, using the construct in a clinical setting, ASCs are expected to be able to differentiate into adipocytes within the fibrin matrix to form new adipose tissue. Therefore, we tested the potential of ASCs to differentiate in the fibrin matrix and evaluated the lipid accumulation in ASCs 28 days after induction of differentiation. Cells were incubated with Preadipocyte Differentiation Medium for 5 days. For the next 24 days cells were cultured in Adipocyte Nutrition Medium.

**Figure 4:**
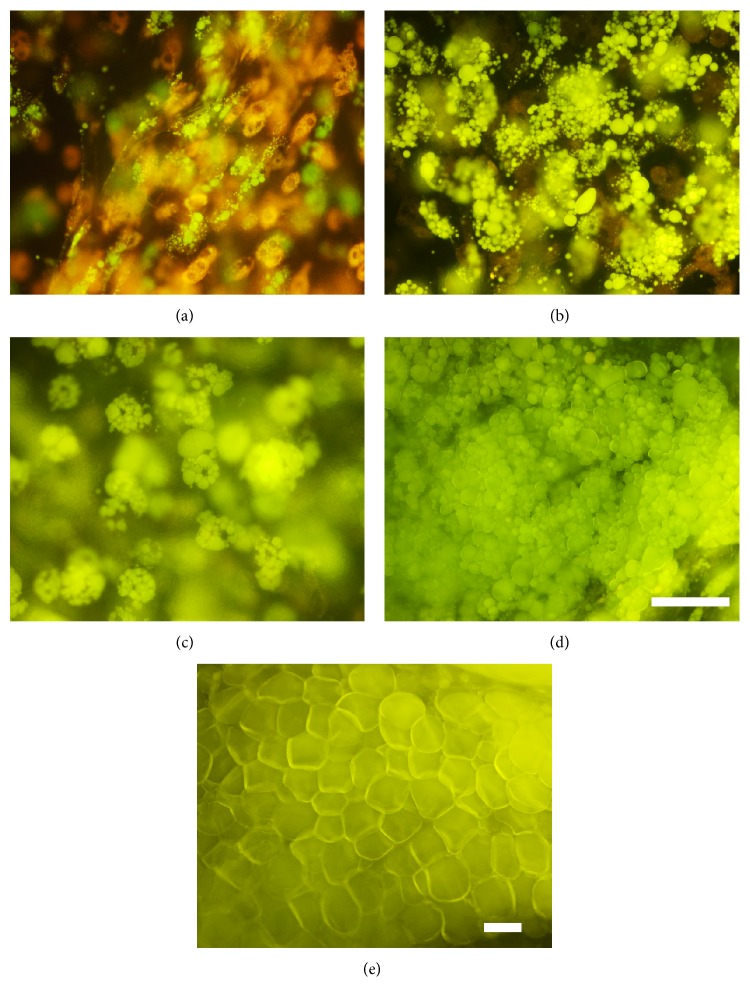
Adipose-derived stem cells differentiating into adipocytes during the time course of 28 days. Differentiation was evaluated by fluorescent AdipoRed staining (a) 7 days, (b) 14 days, (c) 21 days, and (d) 28 days after induction of differentiation. (e) AdipoRed staining of native adipose tissue. Bars represent 100 *μ*m. One representative out of eleven experiments is shown.

On day 7 after induction of differentiation ASCs showed signs of lipid accumulation which increased during the following two weeks (Figures 4(a), 4(b), and 4(c)). On day 28 the majority of the cells in the fibrin hydrogel showed typical adipocyte morphology (Figure 4(d)) similar to native adipose tissue (Figure 4(e)) and filled most of the space in the matrix.

To further evaluate the differentiation potential of ASCs in the fibrin hydrogel we quantified the glycerol release of the cells within the fibrin clots in the supernatant. Undifferentiated ASCs within the fibrin hydrogel were used as control. Additionally, native adipose tissue of the same size as the fibrin matrix was used as positive tissue control.

**Figure 5:**
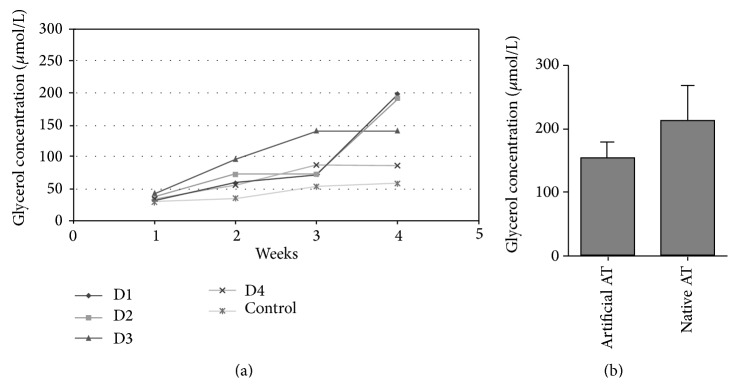
Glycerol concentration (*μ*mol/L) in supernatant of fibrin clots with differentiating ASCs at weeks 1, 2, 3, and 4. A total of *n* = 4 donors (D1–4) are shown. Glycerol concentration of undifferentiated ASCs within the matrix is shown as control. (b) Glycerol release of artificial adipose tissue (artificial AT; *n* = 4) on day 28 compared to fresh, native adipose tissue (native AT; *n* = 4).

Results showed that glycerol release increased up to three times between week 1 and week 4 of adipogenesis (Figure 5(a)). Glycerol concentration of artificial adipose tissue on day 28 was comparable to the concentration measured in the supernatant of native adipose tissue (Figure 5(b)).

A certain variability was observed in terms of differentiation potential of ASCs from different donors. Still, our results demonstrate that ASCs are able to differentiate into adipocytes in a fibrin matrix and therefore are suitable for our three-layered skin substitute.

## 4. Discussion

In this study we were able to generate for the first time a three-layered skin substitute based on a fibrin hydrogel. By incorporating ASCs and mature adipocytes but also fibroblasts into the matrix and seeding keratinocytes on top of the construct, we were able to achieve a skin substitute not only serving as a dermal and epidermal substitute but also including the hypodermis.

In such a construct the main challenge is to generate a layer of adipose tissue replacing the hypodermis. Physical properties are of major importance to mimic the soft character of fat tissue. Mechanical properties of fibrin hydrogel resemble those of adipose tissue to a satisfying extent. In vitro it was already suggested to be suitable for adipose tissue-equivalent formation [13] and because of its biodegradability and biocompatibility we have chosen fibrin hydrogel as a scaffold for our project. Moreover, fibrin hydrogel has already been well characterized and been used successfully as a glue in clinical practice for a long time [21].

In previous studies 3D fibrin matrix of low fibrinogen concentration showed eligible results in adipogenesis of ASCs [13]. Therefore, the fibrinogen component was diluted to 25 mg/mL end concentration in the clot. Additionally, high thrombin concentrations have a negative influence on different cell types, for example, on keratinocytes [22] or endothelial cells and fibroblasts [10]. Consequently, the thrombin component was diluted as well to an end concentration of 2.5 IU/mL in the clot.

Our results show that ASCs and fibroblast were viable and showed physiological morphology in the fibrin hydrogel. Both cell types proliferated well by showing a significant increase of cells in the construct during the first seven days. Additionally, ASCs were able to differentiate into mature adipocytes during the course of four weeks. On day 28 after induction of differentiation lipid accumulation of differentiated ASCs showed morphological resemblance to native adipose tissue. Due to high donor variability there were big differences in the adipogenic differentiation potential of our ASCs. Still, analysis of the glycerol release rate of ASCs during the differentiation period of four weeks indicated that the hypodermis of our artificial skin substitute mimics native adipose tissue.

A variety of skin substitutes that restore epidermal and dermal structures are currently available on the market. Still, there is a lack of hypodermal tissue substitutes. But especially patients with full thickness skin defects would benefit from such a replacement. Although this study showed promising results in the in vitro settings, where we used skin substitute constructs with the size of 5 mm^3^, the missing vascularization presents a limitation to large scale clinical approaches. Successful applications of skin substitutes are currently limited to tissue less than 2 mm in thickness [23]. Applying thicker substitutes would require a capillary network delivering oxygen and nutrients to the tissue.

A promising approach to vascularization of bioartificial skin presented a study by Sánchez-Muñoz et al. by promoting formation of capillary structures by seeding HUVECs with dermal fibroblasts and ASCs in a fibrin matrix. The extracellular matrix, produced by dermal fibroblasts and ASCs, stimulates cellular growth and proliferation [11]. Further possible developments of this new multilayered model besides working with endothelial cells could be the additional use of proteins like PDGF to stimulate neovascularization [24] or incorporation of melanocytes for UV-protection [25].

## 5. Conclusion

In this study we were able to generate a three-layered skin substitute based on a fibrin hydrogel. ASCs, mature adipocytes, and fibroblasts were incorporated into the matrix. Keratinocytes were seeded on top of the construct and formed an epithelial-like layer. We showed that our artificial hypodermis showed comparable characteristics to native adipose tissue in lipid accumulation as well as in glycerol release. With this work we were able to achieve a skin substitute not only serving as a dermal and epidermal substitute but also including the hypodermis.
